# ‘Willpower’ is not enough: time for a new approach to public health policy to prevent obesity

**DOI:** 10.1186/s12916-023-02803-z

**Published:** 2023-03-15

**Authors:** Susan A. Jebb, Paul Aveyard

**Affiliations:** Nuffield Department of Primary Care Health Sciences, University of Oxford, Oxford, UK

**Keywords:** Obesity, Food, Environment, Policy

## Background

In most high-income countries, we eat too much. Looking around the world, it seems that obesity parallels economic development. Within countries, there is a more mixed picture, but there are few people who actively choose to become overweight. Indeed, weight gain has occurred against a strong cultural pressure to be slim, widespread knowledge of the harms of being overweight, and many people spending time and money actively trying to control their weight. The Health Survey for England suggests almost half the adult population are trying to lose weight at any one time [1]. Yet, despite this, there is a persistent belief among the public and policymakers that the solution is more education and urging people to make the right choices.

Public health prevention policies should not be confused with interventions to support meaningful weight loss treatment for people living with obesity. The latter is best achieved with individual support and specific weight loss programmes. But successful *prevention* of weight primary weight gain or secondary *regain* will also be supported by an environment which does not encourage overconsumption.

## Creating a supportive food environment

How can we do this? Research shows clearly that we overvalue individual decision-making and underestimate the impact of our environment on our behaviour. Consider our study where a supermarket removed chocolate from the most prominent places in selected shops in the run-up to Easter, though the products were still available for sale elsewhere in the store [2]. Prior to the experiment, sales of chocolate in these stores and matched controls, where chocolate was promoted as usual, were similar. In the stores with less prominent positioning, people bought 12% more chocolate in the period before Easter than during the preceding period, while in the stores with (typical) layouts, they bought 31% more. In intervention stores, people put fewer calories in their baskets than control stores. Modern food purchasing environments are set up to maximise profit and not health.

Perhaps we could learn to be hyper-vigilant when shopping, but this requires a level of executive functioning (‘willpower’) that is more that we can reasonably be expected to mobilise at every moment of the day, especially when we are stressed or distracted. Food cues are embedded throughout our environment. Moreover, they prime our behaviour in ways far more subtle than we consciously recognise. In another experiment, children watched food or toy advertisements prior to a cartoon. Later, they were offered a choice of foods to eat. Compared with children who had watched TV without food adverts, children who had seen food advertisements ate more [3]. The same was true when children saw a celebrity on TV who was associated with advertisements for crisps, even though no food was shown [4]. It is unlikely that people perceive that their ‘choice’ in these and other similar experiments had been shaped by the environment. So why does this happen?

The UK Government Foresight report on obesity in 2007 described a reinforcing loop where biological hunger signals dominate over the much weaker satiety cues [5]. What evolved as a survival strategy now leaves us vulnerable to an environment where food is palatable, available, and heavily marketed. Weight gain is an almost inevitable consequence in economically advantaged countries, yet we berate ourselves for lack of willpower. More importantly, our society, expressed through the action of our policymakers, continues to believe that individuals have more control over their choices than is actually the case. This thinking shapes the policy discourse and presents a challenge to the introduction of policies seen as curbing the ‘free market’.

Accepting the need to change our food environment is crucial to making progress towards societies with a healthier weight. This is not something individuals can do alone. In the mid-twentieth century, the food industry worked to provide more food to more people more cheaply following a period when the main threat was undernutrition, but the market needs a reset if it is to deliver for today’s health needs. That probably requires government intervention to encourage and support progressive businesses through a time of change. The soft drink industry levy in the UK provides a good example of what can be achieved. By incentivising reformulation of soft drinks, sugar intake from drinks fell by 30% without decreasing sales [6]. This small change to the environment is predicted to decrease the prevalence of obesity by 0.2–0.9% and the incidence of type 2 diabetes by 0.8–4.4/1000 person-years [7].

Just as no single change in the environment created the high prevalence of obesity, so no one policy can reverse that change. We need to accumulate policies, as we have done in tobacco control, to reverse the environmental changes that have led to overconsumption. This requires sustained action, outlasting the usual political cycles.

But at present, standing between us and a healthier environment is policy inertia. We posit that our strong belief, arising from our daily experience of our self-conscious selves, leads us to consider that our behaviour is consciously governed because we do not perceive the myriad times a day when the environment changes what we do. While we can accept the intellectual argument that advertising works, we tend to view the effects of advertising as much greater on others rather than ourselves [8], and thus, our belief about the conscious drivers of our own behaviour remains intact. Moreover, as citizens or as policymakers, we have a strong moral belief that behaviour change should come from within and that external factors are somehow second-rate ways to change behaviour [9].

## Conclusions

We have strong evidence that fiscal policies, advertising restrictions, and curtailing the availability of unhealthy products changes behaviour [10] and no shortage of policy documents recommending specific interventions to prevent obesity. Yet, only a few are enacted anywhere in the world. Explaining the neurobiological basis of behaviour does not seem to change our view that we are masters of our own destiny but highlighting the everyday experiences when our food ‘choices’ are shaped by the environment may be more persuasive in explaining why the ‘willpower’ model is flawed and, accordingly, open the door to more effective policy action.

## Data Availability

Not applicable.
